# Kannada akshara knowledge in primary school children: measurement of accuracy and reaction time using a cross-sectional study design

**DOI:** 10.12688/f1000research.23653.1

**Published:** 2020-08-12

**Authors:** Mysore Nanda Kumar Usha, Malavika Anakkathil Anil, Shwetha Prabhu, Jayashree S. Bhat, Somashekara Haralakatta Shivananjappa

**Affiliations:** 1 Department of Audiology and Speech-Language Pathology, Kasturba Medical College, Mangalore, Manipal Academy of Higher Education (MAHE), Mangalore, Karnataka, 575001, India

**Keywords:** Akshara knowledge, Alphasyllabary, Akshara identification accuracy, Reaction Time

## Abstract

**Background:** Reading acquisition varies between languages, as languages differ in terms of phonology and orthography. Orthographic knowledge is demonstrated to be crucial in literacy acquisition in most orthographies. The literature on acquisition of orthographic knowledge has focused more on alphabetic orthographies and less is understood in alphasyllabary Kannada language. The present study aimed to understand the akshara knowledge acquisition by measuring akshara identification accuracy and reaction time in typically developing Kannada medium primary school children.

**Methods:** The study consisted of 315 typically developing children, 45 each from Grade I through Grade VII between the age range of 5 years 6 months to 12 years 6 months. The children were assessed for akshara identification accuracy and reaction time using a representative sample of 67 akshara selected at four different levels of complexity: vowels in primary form, consonant with inherent vowels, consonant with vowel diacritics, and consonant clusters. The mean performance was compared between the groups using one-way ANOVA with post-hoc Bonferroni test.

**Results:** One-way ANOVA revealed significant main effect (p≤0.05) of Grade on akshara identification accuracy and reaction time. The post-hoc Bonferroni test revealed that the mean akshara identification accuracy improved significantly (p≤0.05) from Grade I to Grade V and reached a plateau at Grade VI. The reaction time significantly reduced from Grade I to Grade IV and there was no significant change beyond Grade V.

**Conclusion:** The children learning to read alphasyllabary Kannada gain mastery over the majority of aksharas during the initial years of formal schooling, which develops completely by Grade VI. The automaticity in naming akshara develops gradually and reaches a plateau by Grade IV. The present findings indicate that children acquire automaticity in naming akshara early, while the akshara knowledge continues to develop.

## Introduction

Reading is a process of developing a sense of written material through systematic mapping of phonemes to the corresponding grapheme. Language-specific phonological and orthographic knowledge is crucial for successful word decoding. Orthographic knowledge is the information that is stored in memory, which dictates the rules to represent the spoken language in the written form ( Apel, 2011). The role of orthographic knowledge is crucial in literacy acquisition (e.g., Apel *et al.*, 2006; Castles & Coltheart, 2004). The studies conducted in alphabetical languages have reported that orthographic knowledge acts as the strongest predictor of reading success in children ( Bowey, 2005; Caravolas *et al.*, 2013; Hulme *et al.*, 2012; Seymour, 2005). However, there are prevailing arguments about the generalization of theoretical models explaining the reading process and other research findings from alphabetic writing systems to children learning to read in other writing systems ( Karanth, 2003; Nag, 2007). Studies in alphasyllabaries have shown that the developmental pathway of reading is different from children acquiring an alphabetic writing system ( Nag, 2007; Nag & Snowling, 2011; Nag & Snowling, 2012). Alphasyllabaries are phonologically and orthographically different from alphabetic writing systems and share features of both alphabetic and syllabic writing systems. Previous studies have illustrated clear differences between alphabetic and alphasyllabaries languages (e.g., Nag, 2007; Nag, 2014; Tiwari *et al.*, 2011). Alphasyllabaries represent sounds at the level of the syllable called akshara, but have distinct features to indicate sub-syllabic information ( Bright, 1999). These differences explain the variability in the acquisition of orthographic knowledge across orthographies. For example, Seymour *et al.* (2003) reported that children acquired orthographic knowledge by the end of Grade I in alphabetical language such as English, whereas Shu (2003) reported it to be beyond Grade VI in Chinese.

Previous attempts have examined akshara knowledge in alphasyllabary languages, such as Kannada and Malayalam. Nag (2007) investigated children’s acquisition of orthographic knowledge in Kannada, a south Indian alphasyllabary language. The study included a group of 374 primary school children between the age range of 5 to 10 years, studying in regular schools with Kannada language as the medium of instruction. The children were assessed for akshara knowledge twice. The first assessment was done when they were Grade I, II, and III. The follow-up assessment was done after 15 months when the children moved to subsequent Grades. A Syllabograph Recognition Test ( LAB, 2003) was conducted, which consists of 20 Kannada akshara, in which one akshara was a primary vowel, eight aksharas of consonants with inherent vowels, five aksharas of consonants with vowel diacritics, and six aksharas of consonant clusters. The results revealed a significant main effect of time of testing and Grade level with a significant interaction between these variables on akshara knowledge. Akshara mastery was found to continue into elementary school with an accuracy of around 80% during the second assessment. The study noted the differential trend for individual akshara types; consonants with inherent vowels were mastered by the end of Grade I, consonants with vowels diacritics with ligatures and consonant clusters were only partially mastered by Grade II, and this extended beyond Grade IV with a substantial number of children showing less than 50% accuracy even at Grade IV. These findings indicated that orthographic knowledge in Kannada was determined by the orthographic features of akshara.

Another study was conducted by Tiwari *et al.* (2011) in Malayalam, which is another alphasyllabary language. This study investigated akshara knowledge in Grade III children learning to read alphasyllabary. The study included 80 children between the age range of 7–8 years from two Malayalam medium schools in Kerala. The stimuli were similar to the Nag (2007) study, which included 17 Malayalam aksharas, such as a primary form of vowels, consonants with inherent vowels, consonants with vowels diacritics, and consonant clusters. The aksharas was presented in the flashcards one at a time, and the children were asked to name them. The results showed a differential pace of mastery of various akshara types in Malayalam alphasyllabary. Grade III children mastered vowels in primary form and consonants with inherent vowels. Consonants with vowel diacritics showed partial dispersion, whereas the consonant cluster showed extreme dispersion. They also reported that consonant with vowel diacritics was mastered much better than consonant clusters.

Nag (2007) and Tiwari *et al.* (2011) have indicated that the acquisition of akshara knowledge continues beyond Grade III in Malayalam and Grade IV in Kannada. However, there is a paucity of data beyond Grade IV in alphasyllabary languages, specifically in Kannada. Additionally, the studies in alphasyllabary have used a limited sample of aksharas out of a large registry, thus limiting the generalization of the findings. More importantly, the studies in alphasyllabaries have determined mastery by assessing the accuracy of akshara identification. However, fluent reading requires the speed with which the individual aksharas are retrieved from long-term memory. Hence, the speed with which akshara naming can be determined by measuring reaction time for individual aksharas. The speed along with accuracy in recognizing aksharas enables children to decode the words faster, thereby enhancing fluency in reading, resulting in improved comprehension. Hence the present study aimed to develop a comprehensive understanding of akshara knowledge acquisition by measuring both accuracy and reaction time in typically developing Kannada medium primary school children from Grade I to Grade VII within the age range of 5 years 6 months to 12 years 6 months.

## Methods

### Study design and setting

The present research incorporated cross sectional study design with non-random convenient sampling method to recruit the participants. The present research was approved by the Institutional Ethic Committee of Kasturba Medical College, Mangalore, India (IEC KMC MLR 11-18/472). The study was conducted between December 2018 and February 2020. The children from five government schools belonging to urban Mangalore of Dakshina Kannada district affiliated to the Karnataka state board with Kannada as the medium of instruction served as participants. Permission from the school administration and written consent from the parents of the participants was obtained before initiating the study.

### Participants

The study consisted of 315 children, 45 each from Grade I through Grade VII between the age range of 5 years 6 months to 12 years 6 months. The children were divided into seven equal groups based on the Grade and respective age range, as shown in Table 1. The sample size was calculated using the formula n= 2(Zα+Zβ)² σ² / d2 Zα-1.96 at 95% confidence level, Zβ-0.84 at 80% power, based on the study done by Nag (2007). The participants were selected based on the following selection criteria:

**Table 1.** Demographic details of the participants.

Grade	N	Mean age (years. months)	SD (years. months)
Grade I	45	6	0.2
Grade II	45	6.10	0.4
Group III	45	7.9	0.3
Group IV	45	8.11	0.4
Grade V	45	9.9	0.3
Grade VI	45	10.11	0.4
Grade VII	45	12	0.5

***Inclusion criteria***. Children who (a) fit into the age and Grade criteria; (b) belong to regular government Kannada medium school; (c) have either Tulu, Konkani or Beary as a native language (L1); (d) are reported to have average and above-average scholastic performance by the class teachers; and (e) belong to families of middle socio-economic status.

***Exclusion criteria***. Children with (a) significant speech, language, hearing, developmental, intellectual, and neurological disorders, according to the WHO ten-question disability screening checklist ( Singhi *et al.*, 2007); (b) history of class retentions; (c) poor academic performance according to class teachers report; (d) poor attendance according to school records; and (e) a history of change in the medium of instructions.

### Instrumentation and procedure

Kannada akshara knowledge was assessed by measuring akshara identification accuracy and reaction time using a representative sample of a Kannada orthographic inventory. A total of 67 aksharas at four different levels of complexity were selected ( Table 2).

**Table 2.** The representation of sample of Kannada aksharas at different levels.

Levels	Number of akshara’s	Akshara
Level 1 Vowels in Primary form	5	ಆ ಇ ಉ ಔ ಅಂ [a:] [i] [u] [au] [aṃ]
Level 2 Consonant with Inherent vowels	3	ನ ಲ ಡ [na] [la] [da]
Level 3 Consonant with Vowel diacritics	53	ಕಾ ಚಿ ಟೀ ತು ಪೂ ಗೆ ಜೆೀ ಡೆೊ ದೋ ಬಾ [ka:] [tʃi] [ʈi:] [tu] [pu:] [ge] [ʤe:] [ḍo] [do:] [ba:] ಖೀ ಛೀ ಠು ಥೊ ಪೆ ಘೀ ಝೊ ಧಾ ಭಿ [k ^h^i:] [tʃ ^h^i:] [t ^h^u] [t ^h^u:] [pe] [g ^h^e:] [ʤ ^h^o] [d ^h^a:] [b ^h^i] ಯೀ ರು ಲು ವೆ ಶೆೀ ಷೆೊ ಸೋ ಹಾ ಳಿ ಣೀ [ji:] [ru] [lu] [ʋe] [ɕe:] [ʃo] [so:] [ha:] [Ɩi] [ṇi:] ನು ಮೊ ಕೆ ಟೆೀ ಪೊ ಗೋ ಡಾ ಬಿ ಠೆೊ ಫೊ [nu] [mu:] [ke] [ʈe:] [po] [go:] [ḍa:] [bi:] [t ^h^o] [p ^h^o] ಘು ಢೊ ಭೆ ಯೀ ಲಾ ಶಿ ಸೀ ಳು ಣೆ ನೂ [g ^h^u] [d ^h^u:] [b ^h^e] [ji:] [la:] [ɕi] [si:] [Ɩu] [ṇe] [nu:] ಮೇ ಲೊ [me:] [lo]
Level 4 Consonant clusters	6	ಕ್ಕ ಪ್ರ ಡ್ಗ ಕ್ಶೆ ಶ್ಲೋ ಣೆ [kka] [pra] [dga] [kʃe] [ɕlo:] [trai]

To facilitate the akshara naming task, the aksharas from the representative sample were initially typed in Kannada orthography with black colored font in a Microsoft PowerPoint (PPT) using Nudi software v5.0. A constant font type ‘Nirmal Ul’ and font size 250 were maintained. The PPTs were converted as a JPEG image to create an individual akshara image having uniform dimension.

The aksharas were presented visually through a laptop installed with licensed version of Paradigm Experiment software v2.5.0.68, which facilitates data acquisition on akshara naming accuracy as well as reaction time. (DmDX 5.1 software is a freely available alternative that could be used.) The Paradigm Experiment software was programmed to present the individual aksharas randomly but in a fixed order for every participant with 3000ms onscreen stimulus duration and 2500ms inter-stimuli interval. The system was connected to a microphone, which recorded the participant’s vocal responses and provided the reaction time automatically in an Excel document. The reaction time was measured in milliseconds, and is defined as the total time elapsed between the presentation of akshara and the onset of the correct naming. Once the responses were recorded, the accuracy of the correct identification of aksharas was checked manually by listening to the response offline and noting down the corresponding reaction time for further statistical analysis. During the analysis, every accurate naming of aksharas was scored ‘1’ and ‘0’ for inaccurate naming. The reaction time was considered only for the correct responses. The calculated accuracy of the response and the corresponding reaction time were tabulated and subjected to statistical analysis.

The participants were made to sit comfortably in front of the laptop in a classroom with relatively low ambient noise with adequate illumination. The laptop was connected headset with microphone positioned at participant’s eye-level with a constant distance of 1 meter. The participants from each group were tested individually. The participants were instructed to name the aksharas as quickly and correctly as possible. All the participants were familiarized with the task through practice trials before the actual testing.

### Statistical analysis

Statistical analysis was carried out using SPSS software version 17.0. Initially, the raw data were summarized in terms of mean, standard deviation, and range of scores using descriptive statistics. Later, the performance was compared across the Grades using one-way ANOVA with post-hoc pair-wise comparison using the Bonferroni test. Later step-wise linear multiple regression analysis was carried out to investigate the independent contribution of akshara identification accuracy and reaction time in word decoding skills. A statistical significance of p<0.05 was considered significant in the present study.

## Results

**Figure 1.**
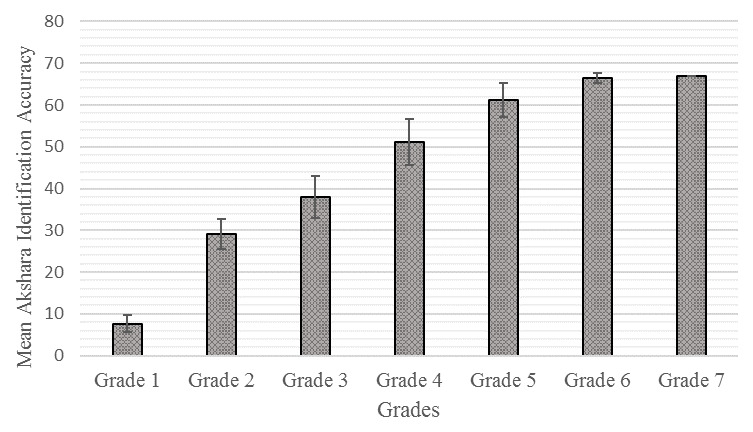
Mean and standard deviation for akshara identification accuracy from Grade I to Grade VII.

**Figure 2.**
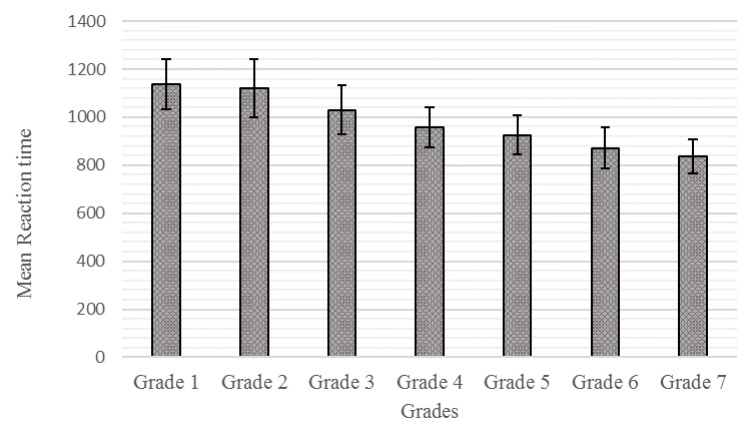
Mean and standard deviation for reaction time from Grade I to Grade VII.

Figure 1 and Figure 2 show the mean and SD for akshara identification accuracy and reaction time across the Grades, respectively.

The result of one-way ANOVA revealed a significant main effect of Grades on akshara identification accuracy [F (6, 308) = 1687.057, p=0.00] and reaction time [F (6, 308) = 69.84, p= 0.00]. Subsequently, a post-hoc pair-wise multiple group comparison was performed using the Bonferroni test, which revealed that the mean performance of Grade I on akshara identification accuracy was significantly lower than higher Grades (p≤0.05). This result was the same for Grades II, III, IV and V in comparison to higher Grades (p≤0.05). However, there was no significant difference between Grade VI and Grade VII (p≥0.05).

The post-hoc pair-wise multiple group comparison of mean reaction time using Bonferroni test failed to reveal any significant difference (p≥0.05) in mean performance between Grade I and II, but Grade I children took significantly more time (p≤0.05) in naming aksharas than all higher Grades. This result was the same for Grades II and III in comparison to all higher Grades (p≤0.05). However, there was no statistically significant difference between Grade IV and Grade V, but the arithmetical mean showed Grade IV took a longer time than Grade V. The performance of Grade IV was significantly lower than Grades VI and VII (p≤0.05). Similarly, there was no significant difference (p≥0.05) noted in the mean performance between Grades V and VII, but the mean performance of Grade V was significantly lower than Grade VII (p≤0.05). There was no significant difference noted (p≥0.05) in the mean performance between Grades VI and VII, which indicated that children from Grades I to IV took significantly greater time to identify the akshara compared to Grades VI and VII who took substantially less time.

## Discussion

The results of one-way ANOVA revealed that accuracy in identifying akshara improves with Grade. On post-hoc pair-wise multiple group comparison, it was observed that the mean performance of children in identifying Kannada aksharas improved significantly from Grade I through to Grade V and reached a plateau at Grade VI, indicating a steady improvement in children’s akshara identification scores. The results showed a developmental trajectory, where children mastered every level of representative aksharas gradually in each Grade, and completely acquired all levels of representative aksharas by Grade VI. These findings are in agreement with results obtained by Nag (2007), who reported that the acquisition of akshara knowledge goes beyond ten years of age (Grade IV). In alphabetical languages, like English, children are reported to master letter knowledge by Grade I ( Seymour *et al.*, 2003) due to the lower number of graphemes compared to alphasyllabary languages, which consists of an extensive number of graphemes and also requires the mastery of a large group of orthographic register and their ligaturing rules ( Nag, 2007). In alphabetic knowledge, the acquisition of letters of the alphabet takes place in a gradual manner, i.e. children learn the letters of the alphabet on the letter by letter basis, rather than learning the entire set at once. Therefore, young children are introduced initially to consonants followed by vowels, as consonants are more consistent in their sounds and hence can be quickly learned compared to vowels ( Strickland, 1998). The gradual, steady improvement in the identification of akshara can be attributed to the manner in which aksharas are taught in schools. Generally, aksharas are taught to children in a hierarchical manner where they are introduced to the aksharas based on the level of complexity, i.e. initially, children are introduced to primary vowels and consonant with inherent vowels, followed by consonants with vowel diacritics, and consonant clusters ( Karanth & Prakash, 1996).

The findings of reaction time measurement from the present study revealed that the children from Grade I to IV took a significantly greater time to identify aksharas compared to Grades V, VI and VII, who took significantly less time. This implies that younger children took a significantly longer time in identifying aksharas compared to older groups. The reduction in reaction time can be described as developmental changes in the speed of information processing ( Kail & Salthouse, 1994; Kail, 2008). Studies report that increases in the speed of processing are correlated with age-related improvements in performance on a variety of cognitive tasks, including reading, memory, solving arithmetic problems, finding their way, and reasoning ( Kail, 2004; Kail, 2008). Even though there was an increase with Grades, the performance of reaction time reached a plateau at Grade IV, and there was no significant difference noted in time taken to name aksharas above Grade IV. The occurrence of plateau in reaction time can be attributed to familiarity of the curricular material to which children are exposed frequently during primary school years. For example, most of the text introduced to Grade I children included words which are formed by primary vowels and consonant with inherent vowels, while in older grades, children are introduced with the words with diacritic markers and clusters, thus making them more familiar to those levels of complexity. As a result, children are more familiar with the aksharas which makes naming speech reach a constant level, but exploring and learning new aksharas continues across grades. Thus, automaticity in naming aksharas develops prior to akshara identification accuracy, which continues to develop above Grade IV and is mastered by Grade VI.

## Conclusion

Children learning to read alphasyllabary Kannada language gain mastery over majority of the aksharas during the first five years of formal schooling and this develops completely by Grade VI. The automaticity in naming akshara develops gradually and reaches a plateau by Grade IV itself. The present findings indicate that children acquire automaticity in naming akshara early, whereas the akshara knowledge continues to develop.

## Data availability

### Underlying data

Harvard Dataverse: Akshara knowledge in Kannada language, https://doi.org/10.7910/DVN/7TJIMS ( Shivananjappa, 2020).

Data are available under the terms of the Creative Commons Zero "No rights reserved" data waiver (CC0 1.0 Public domain dedication).
