# Supplementary material for: Kannada akshara knowledge in primary school children: measurement of accuracy and reaction time using a cross-sectional study design
Source: F1000Res. 2020 Aug 12;9:978. [Version 1] doi: 10.12688/f1000research.23653.1 (PMC7533732; doi:10.12688/f1000research.23653.1)
Supplement: Peer review history

# Peer review: Reviewer response for version 1

**DOI:** 10.5256/f1000research.26100.r72285

**Published:** 2020-10-02

**Authors:** Sangamanatha Ankmnal-Veeranna

**Affiliations:** 1 School of Communications and Sciences and Disorders, Western University, London, Canada

The manuscript examines the *akshara* knowledge in primary school children. The *akshara* knowledge acquisition was examined by measuring the *akshara* identification accuracy and reaction time. Study participants included typically developing children between the ages of 6 to 12 years.  The introduction is well written. The authors have briefly explained about the alphabetic and alphasyllabaries languages. Authors have also reported previous studies that were carried out to examine the development of *akshara *knowledge in Kannada and Malayalam language. The authors have discussed the findings of their study.  

 The following are my comments regarding the manuscript.

**Introduction:**

 I have some questions regarding the justification for the need for the study. Why do authors think that it is important to examine the acquisition of *akshara* knowledge beyond grade IV?

**Method:**

 How long was the experiment?

 Did children receive feedback for their responses?

 The authors have mentioned that scoring was carried out by listening to the recorded sample. Who carried out the scoring?

**Statistical Analysis:**

 Authors have mentioned that they carried out the multiple linear regression analysis on the data. But I do not see those analyses in the result section.

 Is there any association between the reaction time and the accuracy of *akshara *identification?

**Discussion:**

 Why the knowledge of maturation of *akshara *identification is important? Are their clinical implications?

Is the work clearly and accurately presented and does it cite the current literature?

Yes

If applicable, is the statistical analysis and its interpretation appropriate?

Yes

Are all the source data underlying the results available to ensure full reproducibility?

Yes

Is the study design appropriate and is the work technically sound?

Yes

Are the conclusions drawn adequately supported by the results?

Yes

Are sufficient details of methods and analysis provided to allow replication by others?

Yes

Reviewer Expertise:

Development and electrophysiology

I confirm that I have read this submission and believe that I have an appropriate level of expertise to confirm that it is of an acceptable scientific standard.

# Peer review: Reviewer response for version 1

**DOI:** 10.5256/f1000research.26100.r69436

**Published:** 2020-09-03

**Authors:** Pradeep Yuvaraj

**Affiliations:** 1 Department of speech pathology and audiology, National Institute of Mental Health and Neurosciences, Bangalore, Karnataka, India

The article begins with a well writen introduction where in the justification for the present study is noted. The introduction though states that there are literature available which suggests that the reaction time gets better with age, the present study aims to find the plateau for reaction time. The research question is clear and unambiguous.

 The method section describes the test material, inclusion and exclusion criteria and the mode of stimulus presentation comprehensively. 

 The statistical analysis for the group and between group are well described in the results chapter. The demographics are well represented graphically.

 The discussion derives the inference from the available literature with proper justification for the results from the present study. However the results should have thrown up little more information on neurobiology for processing with age.

Is the work clearly and accurately presented and does it cite the current literature?

Yes

If applicable, is the statistical analysis and its interpretation appropriate?

Yes

Are all the source data underlying the results available to ensure full reproducibility?

Yes

Is the study design appropriate and is the work technically sound?

Yes

Are the conclusions drawn adequately supported by the results?

Partly

Are sufficient details of methods and analysis provided to allow replication by others?

Yes

Reviewer Expertise:

Neuroaudiology, Electrophysiology , Adult communication disorders

I confirm that I have read this submission and believe that I have an appropriate level of expertise to confirm that it is of an acceptable scientific standard.
